# Association between Vitamin D Level and Sensorineural Hearing Loss in Adults: Systematic Review and Meta‐Analysis

**DOI:** 10.1002/fsn3.71721

**Published:** 2026-04-04

**Authors:** Ahd Mortada Awad Mohmed, Tasnim Omer Mohamed Elawad, Abrar Abd Elmohaimin Ali Mohammed, Tanzeel MohamedSalih, Mohamed H. Elbadawi, Yousef Mohamed Ahmed Alslawy

**Affiliations:** ^1^ Faculty of Medicine University of Khartoum Khartoum Sudan; ^2^ Faculty of Medicine Mansoura University Mansoura Egypt

**Keywords:** cholecalciferol, sensorineural deafness, sensorineural hearing loss, SNHL, vitamin D

## Abstract

Sensorineural hearing loss (SNHL) is the most common cause of hearing loss. Several studies have explored the association between vitamin D Levels and the incidence of SNHL. This systematic review and meta‐analysis aim to assess the association between vitamin D and the SNHL in adult patients.This meta‐analysis was conducted according to PRISMA guidelines. A comprehensive search was performed through PubMed, Embase, Cochrane, and Scopus on November 16, 2024, for all studies assessing the association between vitamin D levels and SNHL. The risk of bias was assessed using the Newcastle‐Ottawa Scale (NOS). Meta‐analysis was conducted using R software, with heterogeneity being assessed by the I2 statistic. Subgroup analysis was also performed. Five observational studies were included, with a total sample size of 1936 and a mean age of 61.9 years. Vitamin D levels were significantly lower in the SNHL group compared to healthy (MD = −9.50, I2 = 16.9%). The incidence of sufficient vitamin D levels was significantly lower in the SNHL group than in healthy (RR = 0.61, I2 = 85.3%). Subgroup analysis revealed a significantly lower risk ratio of sufficient vitamin D in the Sudden SNHL group than in the other types of SNHL. This meta‐analysis suggests a significant association between vitamin D levels and the incidence of SNHL. Lower vitamin D levels were reported among SNHL patients. Further investigations are needed to account for the confounders of this association using less biased methodologies, such as cohort studies and clinical trials.

**Trail Registration:** CRD420251072753

## Graphical abstract

This graphical abstract illustrates the methodology and key findings of our systematic review and meta‐analysis. We comprehensively searched 4 databases, including 5 observational studies with a total of 1936 participants. The study aimed to compare serum vitamin D levels between adults with sensorineural hearing loss (SNHL) and healthy controls. The analysis demonstrates that lower vitamin D levels are significantly associated with an increased risk of SNHL, particularly sudden SNHL.

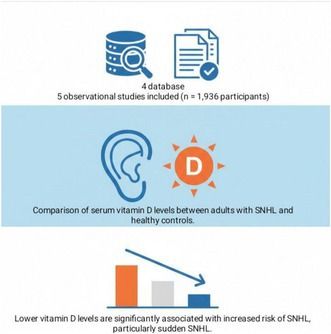

## 1 Introduction

Vitamin D, a fat‐soluble secosteroid, plays a fundamental role in various physiological processes, including calcium and phosphate homeostasis, immune modulation, and cell differentiation and growth. Its most well‐documented function is in the maintenance of skeletal integrity, where it facilitates calcium absorption and bone mineralization. However, beyond its classical role in bone health, emerging evidence suggests that vitamin D may influence other physiological systems, including the auditory system (Kwon 2016).

Sensorineural hearing loss (SNHL), one of the most prevalent forms of hearing impairment worldwide, results from damage to the inner ear or the auditory nerve. The etiology of SNHL is multifactorial, involving genetic, environmental, metabolic, and nutritional components. Recent studies have explored the potential association between vitamin D deficiency and the risk of SNHL. For instance, a cross‐sectional study utilizing data from the National Health and Nutrition Examination Surveys (2001–2006, 2009–2012) found that vitamin D deficiency was significantly associated with bilateral hearing impairment and bilateral SNHL in older adults, especially at lower speech frequencies (Bigman 2022).

Similarly, a retrospective study conducted among children with vitamin D deficiency reported that 53.4% had some degree of hearing loss, with 17% experiencing moderate to profound hearing loss. This study also highlighted a link between hypocalcemia, often a consequence of vitamin D deficiency, and more severe auditory impairment (Mehta et al. 2020).

A meta‐analysis and systematic review published in The Egyptian Journal of Otolaryngology on 17 February 2022 concluded that vitamin D deficiency is associated with several ear‐related conditions, including otitis media, benign paroxysmal positional vertigo, Meniere's disease, tympanosclerosis, and otosclerosis. However, this review did not provide specific data on the association between vitamin D deficiency and SNHL (Salamah et al. 2022).

Despite these suggestive findings, other studies have reported conflicting results. Observations indicated that while low vitamin D levels were linked to low‐ and speech‐frequency hearing loss, the strength of the relationship was insufficient to establish a causal connection. These inconsistencies may stem from heterogeneity in study designs, sample sizes, diagnostic criteria, and methods of assessing vitamin D status (Lee et al. 2024; Szeto et al. 2021).

While the clinical evidence on the relationship between vitamin D deficiency and SNHL remains inconclusive, the biological plausibility of such a link is supported by the presence of vitamin D receptors in the cochlea and its potential involvement in cellular differentiation, calcium regulation, and inflammatory modulation within auditory structures. Nonetheless, more high‐quality, randomized controlled trials are needed to establish a clearer understanding of the role of vitamin D in auditory health (Kwon 2016).

Compounding this clinical concern is the widespread global prevalence of vitamin D deficiency. A comprehensive analysis of 308 studies involving nearly 8 million participants from 81 countries reported that 15.7% of individuals had serum 25‐hydroxyvitamin D levels below 30 nmol/L, 47.9% had levels below 50 nmol/L, and 76.6% had levels below 75 nmol/L. Prevalence rates varied significantly across World Health Organization regions: 35.2% of individuals in the Eastern Mediterranean Region had 25‐hydroxyvitamin D levels below 30 nmol/L, compared to 5.5% in both the African Region and the Region of the Americas. The highest deficiency burden was found in regions with limited sun exposure and in low‐ and middle‐income populations, particularly among women and residents at higher latitudes (Cui et al. 2023).

Given the high prevalence of vitamin D deficiency and its possible impact on auditory function, understanding its potential role in SNHL has substantial public health implications. Addressing vitamin D deficiency through public health initiatives such as dietary supplementation, fortification, and education could offer ancillary benefits in reducing the global burden of hearing loss (Mehta et al. 2020).

## 2 Materials and Methods

This systematic literature review and meta‐analysis was conducted following a protocol based on the transparent reporting of systematic reviews and meta‐analysis (PRISMA) 2020 checklist. It was registered in PROSPERO by the ID: CRD420251072753.

### 2.1 Eligibility Criteria

#### 2.1.1 Inclusion Criteria

Studies were deemed eligible for inclusion if they met the following criteria: observational (cross‐sectional, case–control, cohort) or randomized controlled trials (RCTs) conducted on adult patients (≥ 18 years), duple arm studies that address patients diagnosed with SNHL and healthy individuals with no hearing impairment, serum Vitamin D levels (active Vitamin D or total 25‐hydroxyvitamin D) were reported and compared with levels between patients and controls, and only articles published in English.

#### 2.1.2 Exclusion Criteria

Studies were excluded according to the following criteria: articles that focus on patients with hearing loss not categorized as sensorineural (e.g., conductive or mixed hearing loss), case reports and case series, secondary studies (reviews, meta‐analyses, editorials, commentaries), conference abstracts lacking sufficient data, studies that do not report Vitamin D levels and provide insufficient data for comparison and unpublished studies.

### 2.2 Data Sources and Search Strategy

#### 2.2.1 Data Sources

**FIGURE 1:**
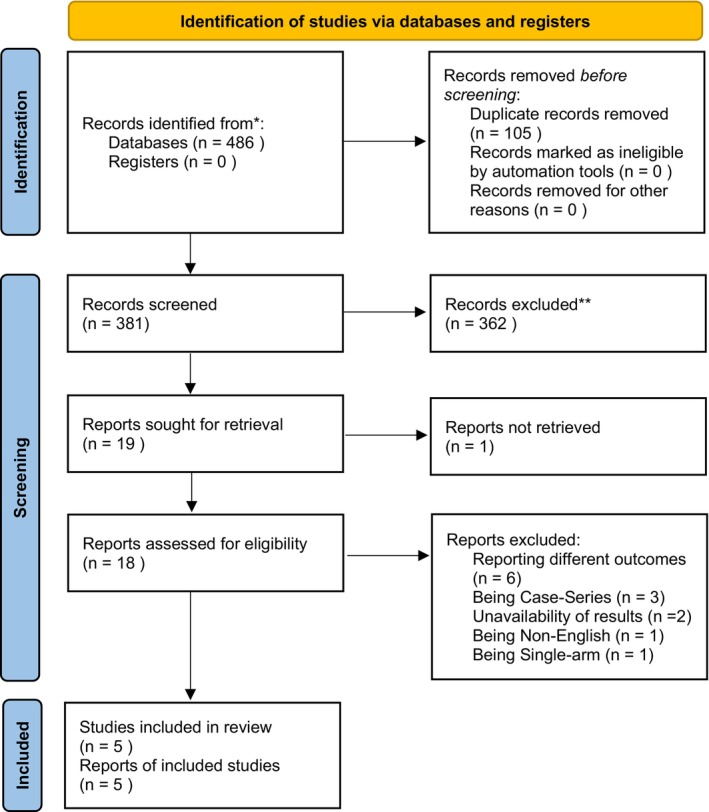
PRISMA 2020 flow diagram illustrating the study selection process. (*) Indicates the number of records identified from specific bibliographic databases. (**) Indicates records excluded based on title and abstract screening criteria.

We conducted a comprehensive database search through PubMed, Embase, Cochrane, and Scopus from inception until November 16, 2024, for all studies assessing the effect of Vitamin D level on SNHL without using any filters (Figure 1).

#### 2.2.2 Search Strategy

We used the same search strategy for all databases: PubMed, Cochrane, Embase, and Scopus: (Vitamin D OR vitamin D2 OR calciferol OR Cholecalciferol OR Hydroxycholecalciferols OR Ergocalciferols OR 25‐hydroxyvitamin D2 OR Dihydrotachysterol OR Viosterol) AND (Neurosensory Deafness OR sensorineural deafness OR sensorineural hearing loss OR Cochlear Hearing Loss OR SNHL).

### 2.3 Screening

The Rayyan website was used as a screening tool (Ouzzani et al. 2016). Two independent reviewers screened titles and abstracts. A third reviewer resolved disagreements. Full‐text articles were also assessed by a fourth author for eligibility based on their methodology and relevant outcomes.

### 2.4 Data Extraction Process and Data Items

Data were extracted using a Google spreadsheet by two authors and double‐checked by a third one. Extracted data included: study characteristics (authors, publication year, country, study design and sample size), participant details (Number of SNHL patients and control, mean age, male percentage, diagnostic criteria for SNHL and its severity), details about Vitamin D assessment (Type assessed, measurement method, deficiency cut‐off points and serum levels for participants) and reported outcomes (primary and secondary outcomes). The primary outcome was serum vitamin D level, while secondary outcomes were: incidences of sufficient vitamin D, incidences of insufficient vitamin D and incidences of deficient vitamin D.

For papers that provided separate means and Standard deviations for patients and another one for controls, we used the StatsToDo website to combine them (CombineMeanSD 2025). We also converted Vitamin D levels when reported as ng/ml to nmol/L by multiplying it by 2.5. When the number of participants with different levels of Vitamin D (sufficient, insufficient, and deficient) was written as a percentage, we converted it manually according to the total participants.

In the study by Hosseini et al. (2020) serum vitamin D was reported as 10 mg/dL in the results section. This value is physiologically implausible and inconsistent with established vitamin D reference ranges. Considering the internal inconsistency within the article and the fact that interpreting the value as ng/dL results in a concentration of 25 nmol/L after unit conversion—which closely aligns with values reported in comparable studies—we considered this discrepancy to represent a typographical unit reporting error. Accordingly, the value was interpreted as ng/dL and converted to nmol/L for consistency across the meta‐analysis.

### 2.5 Risk of Bias Assessment

We utilized the Newcastle–Ottawa quality assessment scale (NOS) (Ottawa Hospital Research Institute 2025) and its adopted version for cross‐sectional analysis (Nunez et al. 2023) to assess the risk of bias across our studies. Two authors used the tool to analyze the studies, and a third author resolved the conflicts. We used seven stars or more as the cut‐off value for a good‐quality study.

### 2.6 Data Synthesis and Analysis

Meta‐analysis was conducted using R software version 4.4.2 (R Studio version 2024). Meta packages were used for all analysis. The effect size measured was the mean difference (MD) for continuous outcomes and the risk ratio (RR) for binary outcomes. Heterogeneity was assessed using I^2^ statistics (I^2^ > 50% considered significant). When significant heterogeneity was present, we applied a random‐effect model; as it assumes that the true effect size varies across studies due to clinical or methodological differences. In contrast, when heterogeneity was low, we used the fixed‐effect model which assumes that all included studies share a common true effect size. The choice of model influences the weighting of individual studies and the width of confidence intervals, with random‐effects models typically producing wider confidence intervals and more conservative estimates. Subgroup and sensitivity analysis were performed in case of significant heterogeneity. A subgroup analysis was conducted based on the type of SNHL, age of the participants, and deficiency cut‐off point to explore potential variations in effect sizes. Sensitivity analysis was performed using the MetaInf package in R to test the robustness of the results. The meta‐analysis results were presented visually using forest plots, with calculated risk ratios, mean differences, and corresponding 95% confidence intervals for each outcome. In all tests, the level of significance used was 0.05.

## 3 Results

### 3.1 Baseline Characteristics

The total number of included studies was five, all of which are cross‐sectional studies except Zheng et al. (2023), a case–control study. The total sample size in this meta‐analysis was 1936 participants, distributed into 1214 for the SNHL group and 722 for the control group. The mean age for the participants included in all studies was 61.9 years, except for the (Hosseini et al. 2020) study, which did not report the mean age. The type of SNHL loss in all studies was Sudden Sensorineural hearing loss (SSNHL) except for Lee et al. (2024) study, which was ARHL, and Hosseini et al. (2020) study, which did not specify the type. The majority of studies defined deficiency as a vitamin D level less than 30 nmol/L, except Lee et al. (2024) and Zandi et al. (2023) studies, which used 50 nmol/L as a cut‐off point (Table 1).

**TABLE 1:** Baseline characteristics of included studies.

Study	Study design	Total sample size	N of SNHL group	N of control group	Age (mean ± SD)	Vitamin D level (SNHL) (mean ± SD) (nmol/l)	Vitamin D level (control) (mean ± SD) (nmol/l)	Type of hearing loss	Vitamin D deficiency cut‐off	NOS assessment
Lee et al. 2024	Cross‐sectional	1104	651	453	71 ± *N*/A	N/A	N/A	ARHL	Deficiency < 50 nmol/l	bad quality (6 stars)
Zheng et al. 2023	Case control	464	310	154	44.54 ± 11.40	38.50 ± 12.11	47.63 ± 10.32	SSNHL	Deficiency < 30 nmol/l	Good quality (7 stars)
Ghazavi et al. 2020	Cross‐sectional	68	34	34	49.19 ± 15.74	48.12 ± 23.86	64.17 ± 27.98	SSNHL	Deficiency < 30 nmol/l	bad quality (6 stars)
Zandi et al. 2023	Cross‐sectional	100	50	50	50.66 ± 16.43	66.27 ± 36.04	83.64 ± 35.47	SSNHL	Deficiency < 50 nmol/l	bad quality (5 stars)
Hosseini et al. 2020	Cross‐sectional	200	169	31	N/A	N/A	N/A	SNHl	Deficiency < 10 mg/dl^a^	bad quality (5 stars)

Abbreviations: ARHL, Age‐related hearing loss; N/A, Not available; NOS, Newcastle—Ottawa quality assessment scale; SD, Standard deviation; SNHL, Sensorineural hearing loss; SNHL, Sudden sensorineural hearing loss.

^a^ It is reported as 10 mg/dL in the study results; however, this contradicts both the paper itself and the known measurement and normal levels of vitamin D. Therefore, we consider it to be measured in ng/dL, and so convert it to 25 nmol/L as this measurement best aligns with the meta‐analysis results.

### 3.2 Risk of Bias Assessment

According to the NOS tool, almost all studies have bad quality except the (Zheng et al. 2023) study, which was a case–control study and has good quality. All studies had 6 stars or below, except the (Zheng et al. 2023) study, which had 7 stars (Table 1).

### 3.3 Vitamin D Level

**FIGURE 2:**
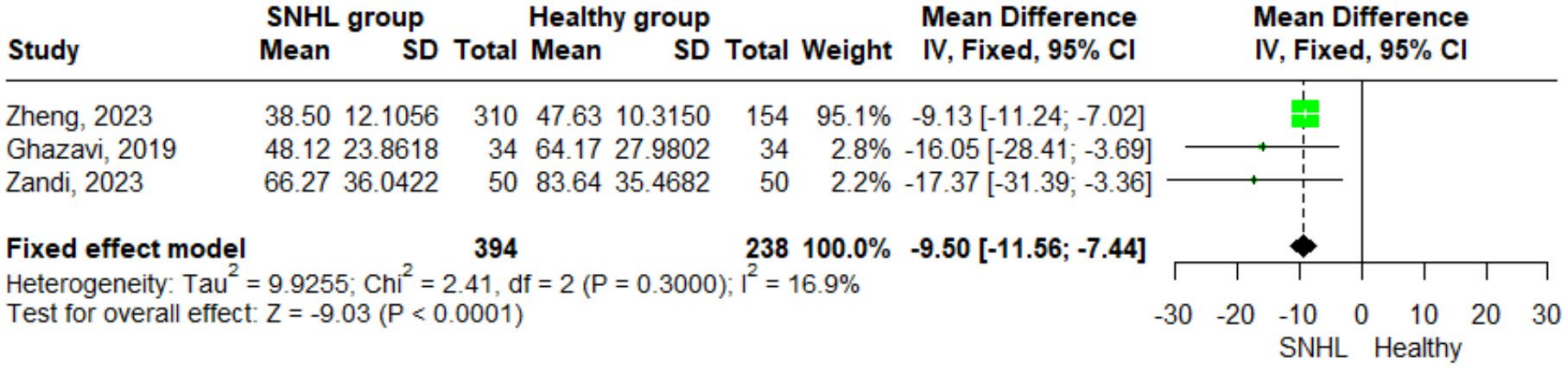
Forest plot comparing mean vitamin D levels (continuous variable) between the SNHL group and the healthy control group using a fixed‐effect model. The green squares represent the mean difference (MD) for each individual study, with the size of the square proportional to the study's weight in the meta‐analysis. Horizontal lines indicate the 95% confidence intervals (CI). The black diamond represents the pooled mean difference.

In this analysis, we only included the studies that reported the mean and standard deviation of vitamin D levels, which were found to be three studies. The fixed‐effect model was performed, and the pooled effect size using Mean Difference (MD) was found to be −9.50 (95% C.I. = −11.56; −7.44, *p* < 0.0001). No significant heterogeneity has been detected (I^2^ = 16.9%; p‐value = 0.300). These results indicate that the vitamin D level is significantly lower in the SNHL group by 9.5 nmol/L than in the control group (Figure 2).

### 3.4 Sufficient Vitamin D Level

**FIGURE 3:**
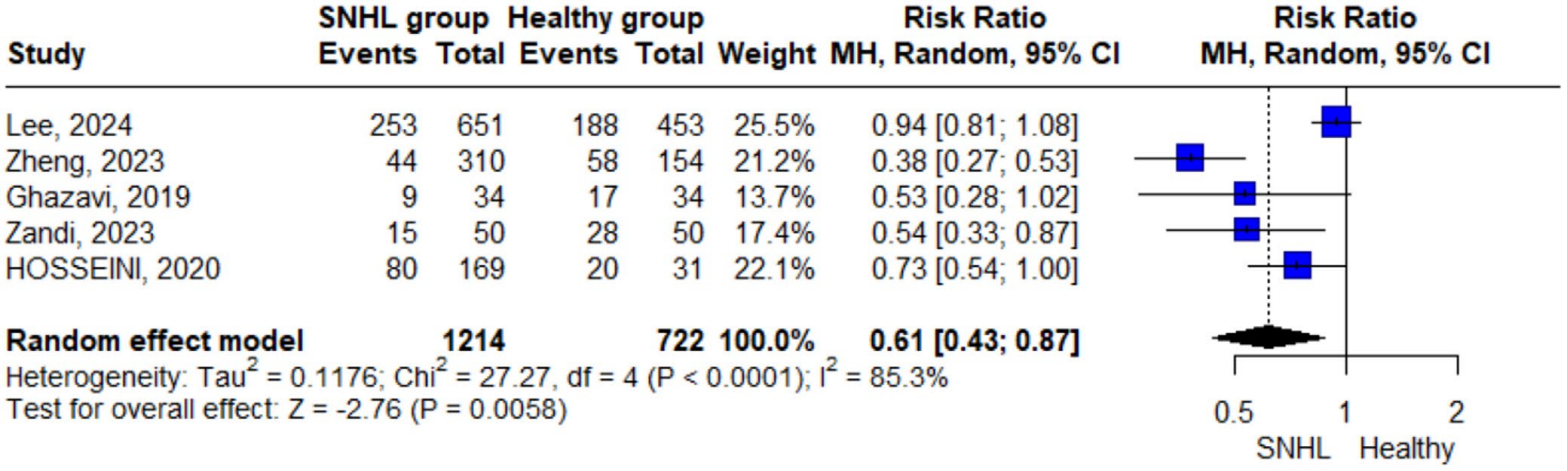
Forest plot showing the risk ratio for having sufficient vitamin D levels in the SNHL group compared to the control group using a random‐effect model. “Events” refers to the number of participants identified as having sufficient vitamin D levels in each group. Blue squares represent the risk ratio for individual studies, and the black diamond represents the pooled risk ratio.

The random effect model was applied for the number of patients with sufficient vitamin D levels, and the pooled effect size for all five studies was 0.61 (95% − CI = 0.43; 0.87, p‐value = 0.006), indicating that the SNHL group was significantly less likely to have sufficient vitamin D levels than the control group. However, the significantly higher heterogeneity (I^2^ = 85.3%; *p* < 0.0001) indicates considerable between‐study variability, suggesting that the true effect size may differ across populations and study settings. Therefore, the pooled estimate should be interpreted with caution (Figure 3). The sensitivity analysis was performed to investigate the source of heterogeneity; however, removing each single study did not eliminate the heterogeneity (Figure S1). Subgroup analysis was performed based on the type of SNHL, age of the participants, and the deficiency cut‐off point. While heterogeneity was low in the SSNHL subgroup, considerable heterogeneity persisted in the age subgroups (I^2^ = 85.3%) and the “Other” types of SNHL subgroup (I^2^ = 49.8%) and the deficiency cut‐off point.

Using a fixed‐effect model, subgrouping based on the type of SNHL showed a significantly lower risk ratio of sufficient vitamin D levels in the SSNHL subgroup than in other types of SNHL subgroup (RR = 0.43 vs 0.91, *p* < 0.0001). There was no significant difference between patients and controls in the other types of SNHL subgroup, while patients in the SSNHL subgroup were significantly less likely to have sufficient vitamin D levels than the controls (RR = 0.43, p‐value < 0.0001).

Regarding subgroup analysis based on age, a subgroup of studies including patients aged < 65 years demonstrated a significantly less likely to have sufficient vitamin D levels than the other subgroups (*p* < 0.0001). Subgroup for studies that included participants without age restriction or aged < 65 years showed a significantly lower incidence of sufficient vitamin D levels among the SNHL patients compared to controls (RR = 0.67, *p* = 0.0058, I^2^ = 0%) and (RR = 0.42, *p* < 0.0001, I^2^ = 25.1%), respectively. Only one study was included in the participants aged > 65 years subgroup.

**FIGURE 4:**
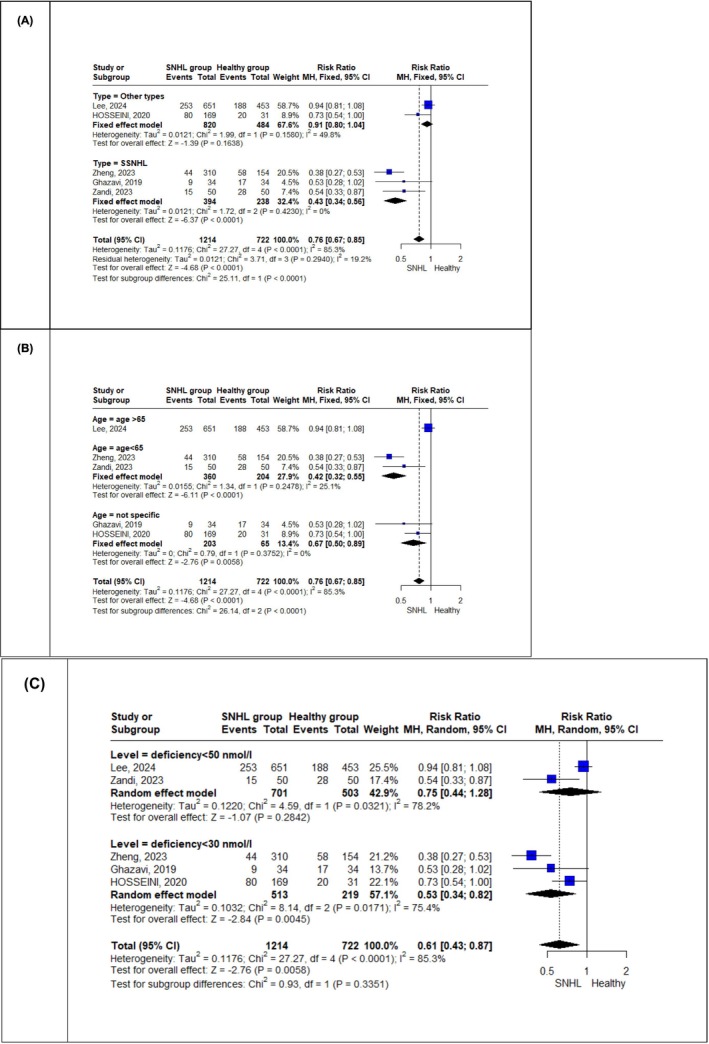
Subgroup analyses for sufficient vitamin D levels. (A) Analysis stratified by the type of SNHL (SSNHL vs. other types). (B) Analysis stratified by participant age (< 65 years vs. > 65 years). (C) Analysis stratified by the laboratory cut‐off point used to define vitamin D deficiency (< 30 nmol/L vs. < 50 nmol/L).

The subgroups based on deficiency cut‐off points were: studies that used < 50 nmol/L as the cut‐off point, and studies that used < 30 nmol/L. A significantly lower incidence of sufficient vitamin D levels was observed in the SNHL patients among studies using < 30 nmol/L as the deficiency cut‐off point (RR = 0.53, *p* = 0.005), compared to controls. In contrast, studies that used < 50 nmol/L showed no significant difference between the SNHL patients and control groups (RR = 0.75, *p* = 0.28). However, no significant difference was observed between the subgroups (*p* = 0.34), and both subgroups showed no significant change in heterogeneity (I^2^ = 78.2%, *p* = 0.032; and I^2^ = 75.4%, *p* = 0.017, respectively) (Figure 4).

### 3.5 Insufficient Vitamin D Level

**FIGURE 5:**
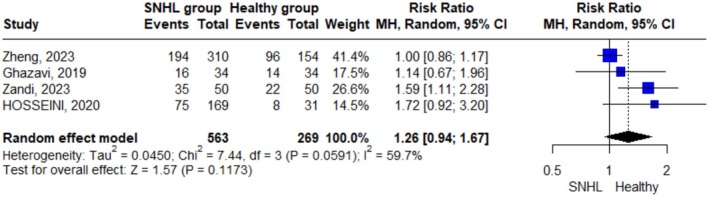
Forest plot describing the analysis of insufficient vitamin D levels using a random‐effect model. “Events” indicates the number of participants with insufficient vitamin D.

Only 4 studies reported the number of patients with insufficient vitamin D levels and were included in the analysis. We found that there was no significant difference in the incidence of insufficient vitamin D level between the SNHL group and the control group as indicated by the pooled effect size results using a random effect model (RR = 1.26; 95% C.I. = 0.94, 1.67; *p*‐value = 0.117) (Figure 5). Significant heterogeneity was noted (I^2^ = 59.7%; *p* = 0.059), so a sensitivity analysis was performed to investigate the source of heterogeneity; however, the removal of each single study did not eliminate the heterogeneity (Figure S2).

### 3.6 Deficient Vitamin D Level

**FIGURE 6:**
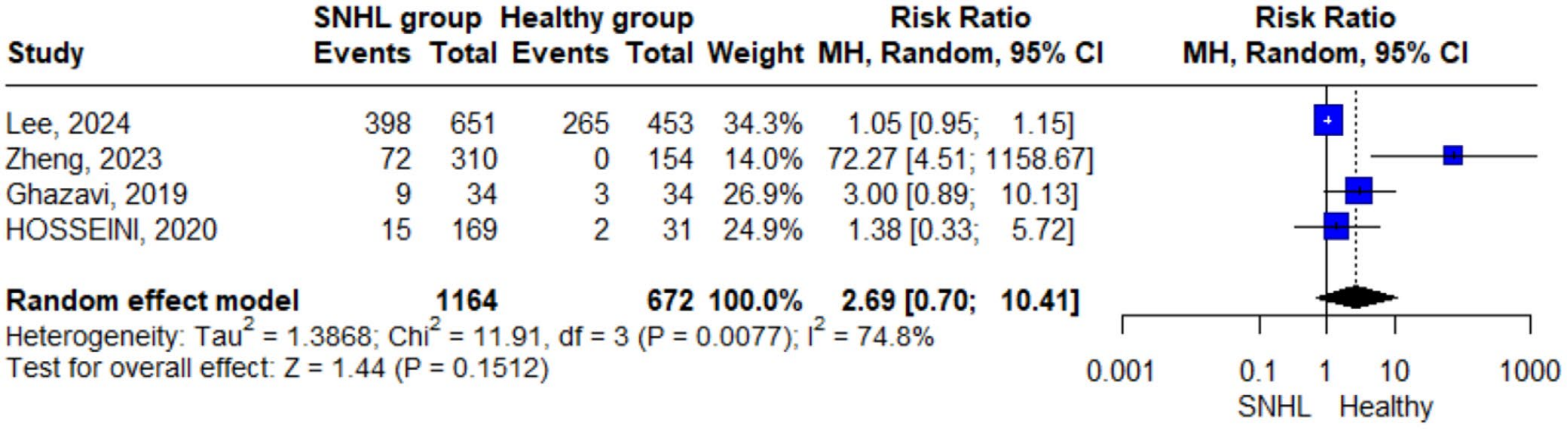
Forest plot describing the analysis of deficient vitamin D levels using a random‐effect model. “Events” indicates the number of participants with vitamin D deficiency.

Only four studies reported the number of patients with deficient levels of vitamin D and were included in the analysis. We found that there was no significant difference in deficient vitamin D level incidences between the SNHL group and the control group as indicated by the pooled effect size results using the random effect model (RR = 2.69; 95% CI = 0.70, 10.41; *p*‐value = 0.151) (Figure 6). However, due to significant heterogeneity (I^2^ = 74.8%; *p*‐value = 0.008), these results suggest substantial variability between studies, and thus the pooled estimate should be interpreted cautiously. A sensitivity analysis was performed to investigate the source of heterogeneity. However, removing each single study did not eliminate the heterogeneity (Figure S3).

## 4 Discussion

Vitamin D has gained attention for its potential role in auditory function, particularly in relation to sensorineural hearing loss (SNHL). As a regulator of calcium metabolism and neuroprotection, vitamin D may influence cochlear health and neuronal signaling. This meta‐analysis examines the association between vitamin D levels and SNHL, highlighting recent findings on its impact on hearing loss risk, severity, and recovery.

Emerging studies suggest that low vitamin D levels may be associated with several otological conditions beyond sensorineural hearing loss (SNHL), including tinnitus, benign paroxysmal positional vertigo (BPPV), otitis media, and Bell's palsy. For instance, in patients with tinnitus, an auditory disorder sharing pathophysiological features with SNHL, vitamin D levels were significantly lower than in healthy controls (49.65 ± 18.83 nmol/L vs. 68.58 ± 22.13 nmol/L, *p* < 0.0001), and deficiency was associated with increased symptom severity (Nowaczewska et al. 2021). Similarly, vitamin D deficiency has been implicated in higher recurrence and risk in BPPV, otitis media, and Bell's palsy (Hamayal et al. 2024). Focusing specifically on SNHL, this meta‐analysis examined the association between serum vitamin D levels and the risk and recovery from SNHL. When vitamin D was treated as a continuous variable, results showed that SNHL patients had significantly lower levels than controls, with a mean difference of −9.50 nmol/L (95% CI = [−11.56; −7.44], *p* < 0.0001). It is worth noting that this pooled estimate closely mirrors the findings of Zheng et al. (2023), which carried substantial weight in the analysis due to its significantly larger sample size compared to the other included studies. Ghazavi et al. (2020) reported that higher serum vitamin D levels were significantly associated with better recovery in SNHL patients. Patients with complete recovery had substantially higher vitamin D levels than those without recovery (54.48 ± 23.68 vs. 27.83 ± 8.28 nmol/L, *p* = 0.004), and all patients with sufficient vitamin D levels achieved complete recovery. These findings support existing evidence on vitamin D's role in cochlear function and neuroprotection, reinforcing its contribution to auditory preservation through modulation of oxidative stress (Paprocki et al. 2021) and calcium regulation (Hamayal et al. 2024).

However, when vitamin D levels were analyzed categorically (sufficient vs. insufficient), no statistically significant association was observed between insufficiency and SNHL risk (RR = 1.26; 95% CI: 0.944–1.667; *p* = 0.117). While sensitivity analysis did not eliminate heterogeneity (I^2^ = 59.7%), potential confounders such as genetic predisposition, dietary intake, and sun exposure variations may have influenced the results. This discrepancy between continuous and categorical analyses may be due to the limitations of classifying vitamin D into broad categories like “sufficient” or “insufficient,” which can hide small but meaningful differences in actual levels. Also, the variation in cut‐off points used across studies could contribute to inconsistent findings, making it harder to detect a clear association. Similarly, the absence of a significant difference in risk ratio when plotting frequencies of both insufficient and deficient vitamin D categories in meta‐analysis reinforces the limitations of categorical analysis and highlights the potential value of continuous‐level assessment in detecting subtle physiological differences relevant to SNHL. Such variability aligns with prior research. For example, a previous study reported significantly lower mean vitamin D levels in SSNHL patients than controls and noted an increased prevalence of vitamin D deficiency in the SSNHL group (Zandi et al. 2023). Additionally, a systematic review highlighted assay variability and inconsistent cut‐offs across vitamin D studies in ear disorders (Salamah et al. 2022). These findings underscore the limitation of categorizing vitamin D; such cut‐offs may hide small yet meaningful differences in serum levels.

The pathophysiological role of vitamin D in SNHL may be attributed to several interrelated mechanisms. Vitamin D receptors (VDRs) are found in the cochlea, and their activation plays a role in regulating calcium homeostasis, an essential factor in hair cell transduction and synaptic transmission. Deficiency in vitamin D may impair these functions, leading to disrupted neuronal signaling and increased susceptibility to cochlear injury (Hamayal et al. 2024). On a molecular level, vitamin D exhibits neurotrophic properties, including promoting neural stem cell proliferation, differentiation into neurons and oligodendrocytes, and survival (Shirazi et al. 2015). Additionally, vitamin D may exert autocrine or paracrine effects on neural tissues, as evidenced by the widespread distribution of VDRs and 1α‐hydroxylase in the brain (Eyles et al. 2005). Moreover, vitamin D plays a crucial role in calcium absorption and homeostasis, which can affect the conduction of electrical impulses in the inner ear between nerve fibers and hair cells (Skinner et al. 2003). Collectively, these actions support the hypothesis that vitamin D deficiency contributes to both the development and progression of SNHL (Paprocki et al. 2021).

Moreover, the role of vitamin D in oxidative stress regulation has been further explored in patients with SSNHL undergoing hyperbaric oxygen therapy. A recent study found that vitamin D supplementation led to a significant reduction in erythrocyte malondialdehyde (MDA) levels after 14 HBO sessions. MDA is a byproduct of lipid peroxidation and a reliable biomarker of oxidative stress, indicating damage to cell membranes caused by reactive oxygen species. Elevated MDA levels are toxic to cells, including the delicate sensory cells of the cochlea. Therefore, the observed reduction in MDA suggests that vitamin D may exert a protective antioxidant effect, helping to minimize cochlear damage and improve treatment outcomes in SSNHL patients.

In terms of treatment outcomes, vitamin D appears to play a therapeutic role. The significant association between vitamin D levels and recovery rates in SNHL patients, as reported by Ghazavi et al. (2020), supports prior evidence suggesting the role of vitamin D in neuroprotection, cochlear function, and inflammatory regulation (Paprocki et al. 2021). In this context, “recovery” refers to the restoration of auditory function as reflected by improvements in hearing thresholds and is thought to involve biological processes such as repair of neural connections and reduction of inflammation within the inner ear. Only one study addressed the recovery rate as an outcome, which is Ghazavi et al. (2020). In this small study of 34 patients, all patients with sufficient vitamin D levels (*n* = 9) achieved complete recovery (*p* < 0.001), highlighting the importance of adequate vitamin D in therapeutic outcomes (Paprocki et al. 2021).

### 4.1 Subgroup Analysis

Subgroup analyses based on SNHL type, age, and deficiency cut‐off points provided more nuanced insights. The overall analysis showed that individuals with SNHL were significantly less likely to have sufficient vitamin D levels than controls (RR = 0.61, *p* = 0.006), though with high heterogeneity (I^2^ = 85.3%). When subgrouped by SNHL type, this difference was significant only among patients with sudden sensorineural hearing loss (SSNHL), who had a notably lower likelihood of sufficient vitamin D compared to controls (RR = 0.43, *p* < 0.0001). No significant difference was observed in the combined non‐SSNHL group (i.e., all other SNHL types analyzed together due to limited study specific data). Similarly, subgroup analysis by age showed that the difference in sufficient vitamin D levels was significant in studies including participants under 65 years or with no age restriction (RR = 0.43 and 0.69, respectively), but not in the single study focusing exclusively on participants over 65. Although subgrouping helped explain part of the variation, heterogeneity remained in most subgroups except those based on age. These findings highlight the importance of considering SNHL subtypes and demographic characteristics in exploring the potential link between vitamin D and hearing function.

## 5 Limitations

A primary limitation of this meta‐analysis is the small number of eligible studies included. Furthermore, the quality of evidence in some included studies was low, which requires caution in interpreting the pooled results. Additionally, the definition of vitamin D deficiency varies throughout studies, which raises the possibility of bias and makes it more difficult to interpret the findings. The variation in deficient thresholds may result in varying prevalence rates, complicating cross‐study comparisons and affecting the final results. In order to more correctly evaluate the impact of vitamin D in SNHL, future studies must also better account for confounding variables like age, comorbidities (such as diabetes, cardiovascular illnesses), and lifestyle factors. Additionally, the results would be more broadly applicable if the populations were more varied in terms of age, ethnicity, and geography.

## 6 Study Implications

The findings of this meta‐analysis suggest that vitamin D may play an important role in protecting against sensorineural hearing loss (SNHL), especially sudden SNHL. While our pooled analysis confirmed lower vitamin D levels in patients, evidence regarding recovery is currently limited to a single study suggesting that sufficient levels might correlate with better outcomes. This implies that vitamin D screening could be relevant for SNHL patients, though its specific role in improving treatment response requires confirmation through further randomized controlled trials. These results also encourage further research into vitamin D's role in hearing health, using consistent definitions and accounting for factors like age, lifestyle, and underlying health conditions.

## 7 Conclusions

This meta‐analysis shows that individuals with sensorineural hearing loss (SNHL), especially sudden SNHL, tend to have lower vitamin D levels than those without. While no clear link was found when using deficiency or insufficiency categories; however, evidence regarding recovery remains limited, as findings suggesting better recovery with sufficient vitamin D levels are currently based on a single study. These findings suggest that vitamin D may support auditory function through its roles in neuroprotection and calcium regulation. Future research should consider factors like age and health status to better understand this relationship.

## Author Contributions

A.M.A.M.: topic selection and validation, investigation, analysis, manuscript writing and revision, and supervision. T.O.M.E.: topic selection and validation, investigation, manuscript writing and revision, and supervision. A.A.E.A.M.: topic selection and validation, investigation, manuscript writing and revision. T.M: topic selection and validation, investigation, manuscript writing and revision. M.H.E.: topic selection and validation, manuscript writing and revision. Y.M.A.A.: manuscript writing and revision.

## Funding

The authors have nothing to report.

## Ethics Statement

The authors have nothing to report.

## Consent

All authors agreed to publish this manuscript.

## Conflicts of Interest

The authors declare no conflicts of interest.

## Supporting information


**Figure S1:** Sensitivity analysis for sufficient vitamin D levels (leave‐one‐out analysis) performed to investigate the source of heterogeneity.

**Figure S2:** Sensitivity analysis for insufficient vitamin D levels.

**Figure S3:** Sensitivity analysis for deficient vitamin D levels.

## Data Availability

The data that support the findings of this study are available from the corresponding author upon reasonable request.
