# Supplementary material for: Association between Vitamin D Level and Sensorineural Hearing Loss in Adults: Systematic Review and Meta‐Analysis
Source: Food Sci Nutr. 2026 Apr 4;14(4):e71721. doi: 10.1002/fsn3.71721 (PMC13052245; doi:10.1002/fsn3.71721)

Study	RR	95%−CI	P−value	Tau2	Tau	I2
Omitting Lee, 2024	0.53	[0.38; 0.74]	0.0002	0.0656	0.2561	63.2%
Omitting Zheng, 2023	0.73	[0.55; 0.97]	0.0282	0.0475	0.2180	62.6%
Omitting Ghazavi, 2019	0.63	[0.42; 0.94]	0.0232	0.1428	0.3779	88.4%
Omitting Zandi, 2023	0.63	[0.41; 0.96]	0.0299	0.1487	0.3856	88.0%
Omitting HOSSEINI, 2020	0.58	[0.37; 0.89]	0.0139	0.1527	0.3907	89.0%
**Random effects model**	**0.61**	**[0.43; 0.87]**	**0.0058**	**0.1176**	**0.3430**	**85.3%**

Leave−One−Out Meta−Analysis

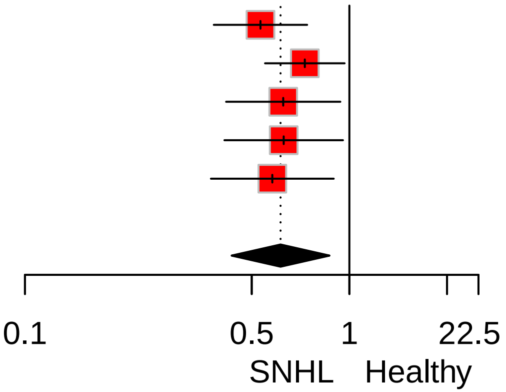

Supplement: Supplementary file 1 — Figure S1: Sensitivity analysis for sufficient vitamin D levels (leave‐one‐out analysis) performed to investigate the source of heterogeneity. [file FSN3-14-e71721-s002.pdf]
