# Supplementary figures and images for: Association between Vitamin D Level and Sensorineural Hearing Loss in Adults: Systematic Review and Meta‐Analysis
Source: Food Sci Nutr. 2026 Apr 4;14(4):e71721. doi: 10.1002/fsn3.71721 (PMC13052245; doi:10.1002/fsn3.71721)

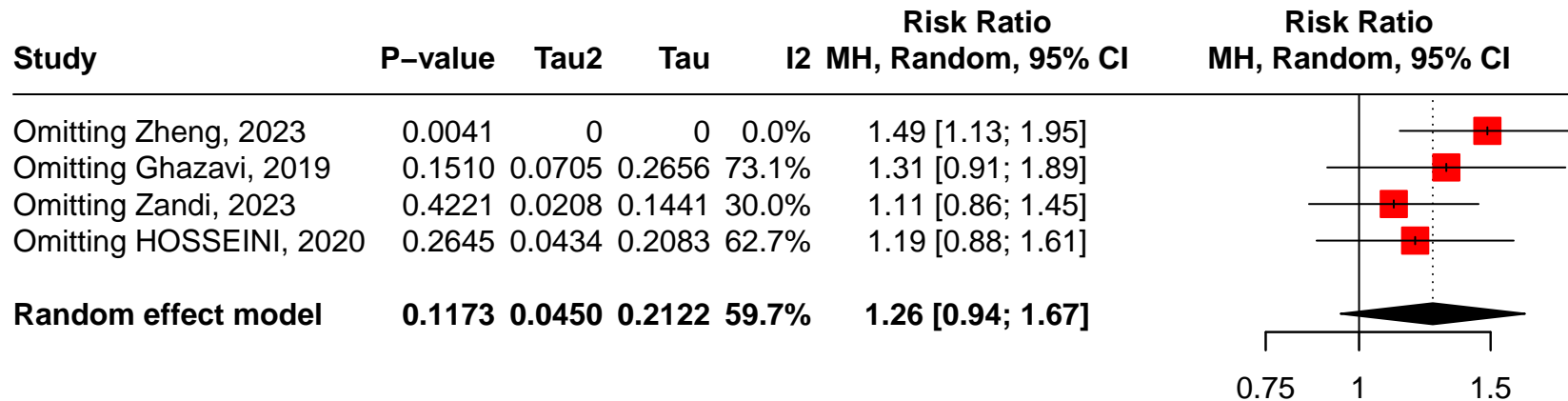

Supplement: Supplementary file 2 — Figure S2: Sensitivity analysis for insufficient vitamin D levels. [file FSN3-14-e71721-s001.pdf]
