# Supplementary material for: Association between Vitamin D Level and Sensorineural Hearing Loss in Adults: Systematic Review and Meta‐Analysis
Source: Food Sci Nutr. 2026 Apr 4;14(4):e71721. doi: 10.1002/fsn3.71721 (PMC13052245; doi:10.1002/fsn3.71721)

| Study | RR | 95%−CI | P−value | Tau2 | Tau | I2 |
|---|---|---|---|---|---|---|
| Omitting Lee, 2024 | 4.86 | [0.66; 35.83] | 0.1208 | 2.2860 | 1.5120 | 67.7% |
| Omitting Zheng, 2023 | 1.32 | [0.72; 2.41] | 0.3728 | 0.1389 | 0.3726 | 33.3% |
| Omitting Ghazavi, 2019 | 3.38 | [0.33; 34.54] | 0.3049 | 3.5271 | 1.8781 | 78.0% |
| Omitting HOSSEINI, 2020 | 4.26 | [0.48; 37.48] | 0.1920 | 3.0712 | 1.7525 | 83.0% |
| **Random effects model** | **2.69** | **[0.70; 10.41]** | **0.1512** | **1.3868** | **1.1776** | **74.8%** |

**Leave−One−Out Meta−Analysis**

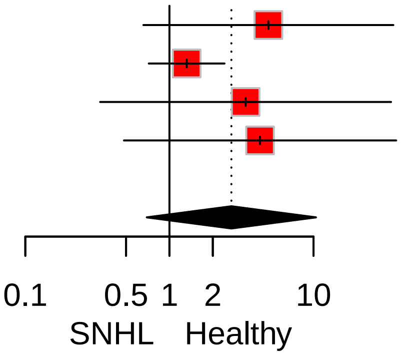

Supplement: Supplementary file 3 — Figure S3: Sensitivity analysis for deficient vitamin D levels. [file FSN3-14-e71721-s003.pdf]
